# Fractional-Order Ultrasonic Sensing for Monitoring Microstructural Evolution in Cementitious Materials

**DOI:** 10.3390/s26010271

**Published:** 2026-01-01

**Authors:** Haoran Zheng, Chao Lu, Xiaoxiong Zhou, Xuejun Jia, Xiang Lv, Zhihan Shi, Guangming Zhang

**Affiliations:** 1 College of Electrical Engineering and Control Science, Nanjing Tech University, Nanjing 211816, China; zhenghaoran@njtech.edu.cn (H.Z.); lc-1206@njtech.edu.cn (C.L.); zhouxx@njtech.edu.cn (X.Z.); 2 China Construction Second Engineering Bureau Co., Ltd., Beijing 100160, China; jxj@njtech.edu.cn (X.J.); jitianyu@njtech.edu.cn (X.L.); 3 School of Electrical and Energy Engineering, Nantong Institute of Technology, Nantong 226001, China; 20256115@ntit.edu.cn

**Keywords:** fractional viscoelasticity, ultrasonic attenuation, hydration, early-age monitoring

## Abstract

Monitoring the early-age evolution of cementitious materials is essential for ensuring the quality and reliability of concrete structures. However, most ultrasonic approaches rely on empirical correlations and lack a physics-based mechanism to describe the continuous viscoelastic transition during hydration. This study proposes a fractional-order ultrasonic sensing framework that couples a fractional Zener viscoelastic model with ultrasonic attenuation theory to quantitatively link microstructural evolution and measured acoustic responses. A custom ultrasonic measurement system was developed to capture real-time attenuation during hydration under different water-cement ratios. Results show that the fractional-order model achieves higher accuracy and robustness than classical integer-order and empirical models. The fractional parameter β serves as a physically interpretable indicator that reflects the transition from viscous-dominated to elastic-dominated behavior and aligns with known hydration development. The proposed framework provides a compact, physics-informed sensing strategy for early-age characterization of cementitious materials and offers potential for intelligent construction and high-end structural monitoring.

## 1. Introduction

The rapid development of high-end infrastructure and equipment—such as shield tunnels, nuclear engineering structures, offshore wind foundations, and large-scale prefabricated components—has created an urgent demand for advanced sensing technologies capable of evaluating material quality and structural performance throughout their entire life cycle [1,2,3]. Cementitious materials, which constitute the core load-bearing components of these systems, undergo complex microstructural evolution during early-age hydration. This evolution directly governs their rheological behavior, stiffness development, defect formation, and ultimately the reliability and service safety of high-end structural equipment. Therefore, real-time and physically interpretable monitoring of the early-age evolution of cementitious materials has become a key enabling technology for intelligent construction and structural health management [4,5,6].

Ultrasonic sensing offers a unique advantage in this context due to its non-destructive nature, high sensitivity to microstructural transitions, and compatibility with embedded and automated monitoring systems [7,8,9,10]. Traditional ultrasonic methods generally rely on empirical correlations between wave velocity, amplitude, or attenuation and the hydration state [1,2,6]. However, such empirical sensing approaches often lack a rigorous physical foundation and provide limited interpretability, especially for the transition from viscous-dominated behavior to viscoelastic–solid behavior during structural formation. The need for model-based, physics-informed ultrasonic sensing has become increasingly evident in high-end engineering applications [3,11].

Recent advances in fractional calculus have demonstrated that fractional-order operators provide a compact yet powerful framework for describing non-local and memory-dependent behavior in complex materials, especially in viscoelasticity and wave propagation [12,13]. In contrast to classical Kelvin–Voigt or Maxwell models, which approximate the relaxation spectrum using a finite number of rheological elements, general fractional viscoelastic models can reproduce broad-band relaxation and power-law-type creep or relaxation with significantly fewer parameters, while maintaining strong physical interpretability [14,15,16,17]. For concrete and other cement-based materials, several recent studies have introduced fractional viscoelastic formulations to capture long-term creep, temperature-dependent deformation and nonlinear damage evolution, showing that fractional models not only achieve higher fitting accuracy but also better reflect the continuous transition of mechanical behavior under varying stress and environmental conditions [16,17]. These works collectively indicate that fractional viscoelasticity is particularly well suited to describing the multi-scale, microstructure-driven rheology of cementitious materials [12,13,14,15].

In parallel, ultrasonic techniques have been extensively employed to monitor the hydration process and early-age property development of cement pastes and concretes, typically by tracking the evolution of ultrasonic velocity, attenuation and backscattered signals [6,7,11,18,19]. Recent developments include omnidirectional or embedded PZT-based transducers and active sensing schemes for identifying key hydration stages and predicting strength development, further highlighting the potential of ultrasound for real-time, non-destructive monitoring in practical structures [19,20]. However, most existing ultrasonic monitoring approaches rely on empirical correlations or simplified linear viscoelastic or poroelastic assumptions for the cement paste matrix, while several studies have applied fractional calculus to model viscoelastic or creep behavior in cementitious materials, the explicit integration of a rigorously derived fractional viscoelastic constitutive law into ultrasonic attenuation and dispersion modeling remains limited [14,21,22,23]. Even in studies where guided waves and fractional viscoelastic parameters are jointly considered—for instance, in layered cement-grouted anchorage systems—the focus remains on parameter identification instead of hydration-induced microstructural evolution [23,24]. Consequently, there is still a clear gap between (i) the rapidly maturing fractional viscoelastic modeling of cementitious materials and (ii) physics-informed ultrasonic sensing models that can exploit fractional parameters as real-time indicators of early-age microstructural evolution, particularly in the context of high-end structural and equipment applications.

To address these limitations, this study introduces a fractional-order ultrasonic sensing framework that couples a fractional Zener viscoelastic model with an ultrasonic attenuation model to quantitatively link hydration-induced microstructural evolution with measurable acoustic attenuation characteristics. A fractional parameter β is introduced to describe the continuous transition from viscous to elastic dominance, providing a physically interpretable sensing indicator. An embedded ultrasonic monitoring system is developed using PZT transducers and a custom excitation–acquisition unit, enabling continuous measurement of attenuation during the early-age period.

In recent years, structural health monitoring (SHM) research for cementitious and reinforced concrete materials has expanded significantly, driven by advances in sensing technologies, non-destructive evaluation (NDE), and data-driven analysis. At the material level, studies on novel cementitious systems—such as self-leveling sand concrete—have investigated the relationships between mixture design, mechanical performance, durability, and non-destructive indicators, demonstrating how material composition and processing influence measurable signals relevant to structural assessment [25]. These efforts highlight the growing role of NDE techniques in linking material properties to performance-oriented monitoring.

At the structural and component levels, machine learning (ML) and deep learning (DL) approaches have been increasingly applied to SHM of concrete and reinforced concrete systems. Recent studies and reviews report that data-driven methods, including neural networks, support vector machines, and ensemble learning algorithms, can significantly enhance damage detection, strain prediction, and condition assessment accuracy in beams, slabs, and bridge structures compared with conventional approaches [26]. In parallel, the integration of piezoelectric sensor networks with deep learning has enabled real-time monitoring of concrete strength and structural state, illustrating the synergy between physics-based sensing and AI-driven interpretation [27]. In addition, research on self-sensing cementitious composites further demonstrates the potential of intrinsically sensing materials for SHM applications [28]. Collectively, these studies establish a broad SHM landscape spanning advanced materials, sensing modalities, and intelligent data analytics, within which the present work positions a physics-informed, fractional-order ultrasonic framework focused on early-age cementitious materials.

Although the present study primarily focuses on the early-age behavior of cement paste at the material scale, this scope is intrinsically aligned with the broader framework of structural health monitoring (SHM). In SHM practice, reliable condition assessment and damage evolution analysis critically depend on the accurate establishment of initial material states and baseline mechanical properties. Early-age hydration governs the formation of load-bearing microstructures, residual stress development, and initial stiffness, which together determine the long-term durability, damage susceptibility, and service performance of concrete structures. Consequently, material-scale sensing during early-age stages provides indispensable baseline information for subsequent component- and structure-level SHM.

From a multi-scale SHM perspective, the proposed fractional-order ultrasonic sensing framework can be naturally extended from laboratory-scale cement paste to structural components and full-scale concrete structures. The physically interpretable fractional parameter β characterizes the intrinsic viscoelastic state of cementitious materials and can serve as a state variable within hierarchical SHM systems, linking microstructural evolution, meso-scale material properties, and macro-scale structural response. When embedded or distributed ultrasonic transducers are deployed in real structures, the evolution of β can be continuously tracked and integrated into digital twin or lifecycle monitoring frameworks, enabling early detection of abnormal material evolution, construction defects, or long-term degradation trends.

The major contributions of this work are summarized as follows:

1. Most existing ultrasonic monitoring approaches for cementitious materials rely on empirical correlations or integer-order viscoelastic/poroelastic assumptions, which provide limited physical interpretability of attenuation mechanisms. The proposed framework fills this gap by embedding a fractional viscoelastic constitutive law directly into the ultrasonic attenuation model, enabling a physics-informed description of material-state evolution;
2. Early-age cement hydration is characterized by a continuous transition from viscous-dominated to elastic-dominated behavior and by distributed relaxation processes across multiple time scales. The fractional Zener model naturally captures this evolving viscoelastic behavior with a compact parameter set, whereas integer-order models with discrete relaxation times are insufficient to represent such continuous transitions;
3. The fractional parameter β serves as a physically interpretable state variable that quantifies the degree of viscoelastic transition during hydration. Unlike conventional ultrasonic indicators (e.g., wave velocity, amplitude, or empirical attenuation coefficients) or isolated rheological parameters, β captures the continuous evolution of intrinsic energy dissipation mechanisms in the cement paste;
4. Compared with previous studies that combine ultrasonic measurements with viscoelastic descriptions in a phenomenological manner, the present work advances the state of the art by directly coupling fractional constitutive modeling with ultrasonic attenuation analysis. This integration improves interpretability and provides a clearer pathway toward multi-scale SHM and digital twin applications.

It should be explicitly noted that the experimental investigations presented in this study are conducted on cement paste only. This choice is intentional, as cement paste constitutes the load-bearing matrix of concrete and governs the intrinsic viscoelastic response during early-age hydration. By excluding aggregates, fibers, and reinforcement, the proposed framework isolates the fundamental coupling between microstructural evolution and ultrasonic attenuation, thereby enabling clear physical interpretation of the fractional parameter β. While this focus inevitably limits the direct generality of the experimental results, it provides a necessary and well-controlled foundation for subsequent extension to more heterogeneous cementitious composites.

The proposed sensing framework provides a foundation for developing next-generation, model-based, embedded ultrasonic systems for quality assessment in high-end structural equipment. It also offers new opportunities for intelligent construction, defect prevention, and digital twins of cement-based infrastructure.

## 2. Theoretical Model

This section delineates the development of a fractional-order theoretical model to precisely characterize the ultrasonic wave propagation in cementitious materials during the early-age hydration process. The limitations of traditional integer-order models are first analyzed to justify the introduction of fractional calculus. Subsequently, the core framework of the fractional-order Zener constitutive model, based on the Caputo derivative, is elaborated, including its frequency-domain expression and the derivation of the ultrasonic attenuation coefficient. The universality of the proposed model is further verified through asymptotic analysis under classical limits.

### 2.1. Limitations of Traditional Models and the Introduction of Fractional-Order Theory

In the domain of viscoelastic energy monitoring for cement-based materials, traditional integer-order rheological models (e.g., the Bingham model $\tau =\tau_{0}+\mu \dot{\gamma }$) are limited because they cannot capture hereditary effects arising from structural evolution and time-dependent properties. Since integer-order derivatives only reflect instantaneous states, they fail to describe the long-range temporal correlations established by the continuously developing hydration network. Therefore, this study introduces fractional calculus theory and constructs a Caputo-type fractional-order constitutive model for viscoelastic characterization. Its derivative is defined as:

(1) $$D_{t}^{\beta }f(t)=\frac{1}{\Gamma (1-\beta )}\int_{0}^{t}{\left(t-\tau \right)}^{-\beta }f^{\prime }(\tau )d\tau \;(0<\beta <1)$$

The Caputo fractional derivative incorporates a kernel ${\left(t-\tau \right)}^{-\beta }$ with inherent memory properties, enabling accurate depiction of the evolving internal structure of cementitious materials—such as the relaxation process after micro-crack closure or cutting damage. Compared with the Riemann–Liouville definition, a major advantage of the Caputo derivative is that its initial conditions are expressed in the same form as classical integer-order models, avoiding ambiguity in specifying initial parameters. The fractional order β introduces dynamic flexibility to the model, supporting its use as a diagnostic parameter for monitoring material evolution. Physically, the fractional kernel describes the cumulative damage and relaxation behavior associated with microstructural evolution.

### 2.2. Fractional-Order Zener Constitutive Model and Its Frequency-Domain Representation

To establish a quantifiable rheological constitutive relationship, the Caputo fractional derivative must be embedded within a physical framework capable of simultaneously characterizing the elastic and viscous behaviors of the material. Accordingly, this study adopts the classical Zener model as the foundational structure for fractional extension. The Zener model, composed of a spring and dashpot in series, can already describe stress relaxation—a key viscoelastic phenomenon of cement-based materials—in its integer-order form. By replacing the integer-order time derivative with the Caputo fractional derivative, the model naturally incorporates power-law memory effects into the stress–strain relationship, thereby yielding an enhanced constitutive equation that captures instantaneous elastic response, long-term relaxation behavior, and the inherent dependence on loading history.

(2) $$\sigma (t)+\tau^{\beta }D_{t}^{\beta }\sigma (t)=E_{\infty }\varepsilon (t)+E_{0}\tau^{\beta }D_{t}^{\beta }\varepsilon (t)$$

Here, $\tau ={\left(\eta /E_{0}\right)}^{1/\beta }$ is the fractional relaxation time, and $E_{0}$ and $E_{\infty }$ denote the instantaneous and long-term elastic moduli, respectively. The parameter β serves as a microstructural diagnostic index: as β→0 the system approaches a viscous-like fluid, whereas as β→1 the system tends toward an elastic solid.

To facilitate the analysis of the model in the frequency domain, a Fourier transform is applied to the fractional constitutive Equation (2). Using the frequency-domain property of fractional derivatives, $F\{D_{t}^{\beta }f(t)\}={\left(i\omega \right)}^{\beta }F(\omega )$, one obtains:

(3) $$\sigma (\omega )[1+{\left(\tau i\omega \right)}^{\beta }]=\varepsilon (\omega )[E_{\infty }+E_{0}{\left(\tau i\omega \right)}^{\beta }]$$

The complex modulus $E^{*}(\omega )$ is defined as the ratio of stress to strain in the frequency domain, i.e., $E^{*}(\omega )=\sigma (\omega )/\varepsilon (\omega )$. Dividing both sides of Equation (3) by ε(ω) (ε(ω)≠0) and substituting the definition of the complex modulus, one directly obtains:

(4) $$\frac{\sigma (\omega )}{\varepsilon (\omega )}\left[1+{\left(\tau i\omega \right)}^{\beta }\right]=E_{\infty }+E_{0}{\left(\tau i\omega \right)}^{\beta }$$

After rearrangement, the explicit expression of the complex modulus is obtained as:

(5) $$E^{*}(\omega )=\frac{E_{\infty }+E_{0}{\left(\tau i\omega \right)}^{\beta }}{1+{\left(\tau i\omega \right)}^{\beta }}$$

The real part (storage modulus $E^{\prime }$) and the imaginary part (loss modulus $E^{\prime \prime }$) of the complex modulus, respectively, characterize the elastic and viscous responses of the material:

(6) E′(ω)=Re[E∗(ω)], E″(ω)=Im[E∗(ω)]

**Figure 1:**
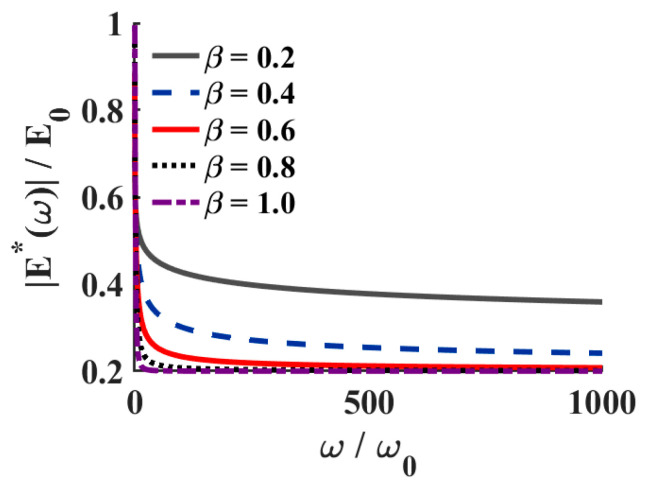
Variation in the magnitude of the complex modulus with normalized frequency.

The frequency-domain representation of the complex modulus $E^{*}(\omega )$ depicts the viscoelastic response of cement paste under acoustic excitation. Figure 1 illustrates the variation in the normalized complex modulus magnitude with frequency for different fractional orders β. As β increases from 0.2 to 1.0, the overall amplitude of the complex modulus gradually stabilizes at a higher level, indicating that the fractional derivative order governs the transition of the material from a viscous-dominated state to an elastic solid-like state. When β is relatively small, the system exhibits pronounced frequency dependence, reflecting stronger memory effects and a dominant viscous contribution. As β approaches 1, the modulus becomes nearly frequency-independent, and the system tends toward an ideal elastic solid. This result confirms that the fractional Zener constitutive model formulated in Equation (5) can effectively capture the frequency-dependent viscoelastic behavior of cementitious materials.

To further reveal how such viscoelastic characteristics affect ultrasonic wave propagation, it is necessary to incorporate the fractional constitutive relation into the wave equation, thereby establishing the linkage between acoustic waves and material parameters.

### 2.3. Derivation of the Ultrasonic Wave Equation and Attenuation Coefficient

This subsection establishes the fractional viscoelastic constitutive description of early-age cement paste. From a physical perspective, the material response during hydration continuously evolves from viscous-dominated to elastic-dominated behavior, accompanied by broad relaxation spectra. The fractional Zener model is adopted here to capture this continuous transition in a compact and physically interpretable manner, forming the basis for subsequent wave propagation and attenuation analysis.

The following assumptions are adopted throughout the theoretical formulation:

(i) The cement paste is treated as an effective homogeneous and isotropic viscoelastic medium;
(ii) Wave propagation is approximated as one-dimensional plane-wave motion;
(iii) Small-strain and linear viscoelastic behavior are assumed;
(iv) Frequency range corresponds to low-frequency ultrasonic excitation relevant to early-age monitoring.

**Figure 2:**
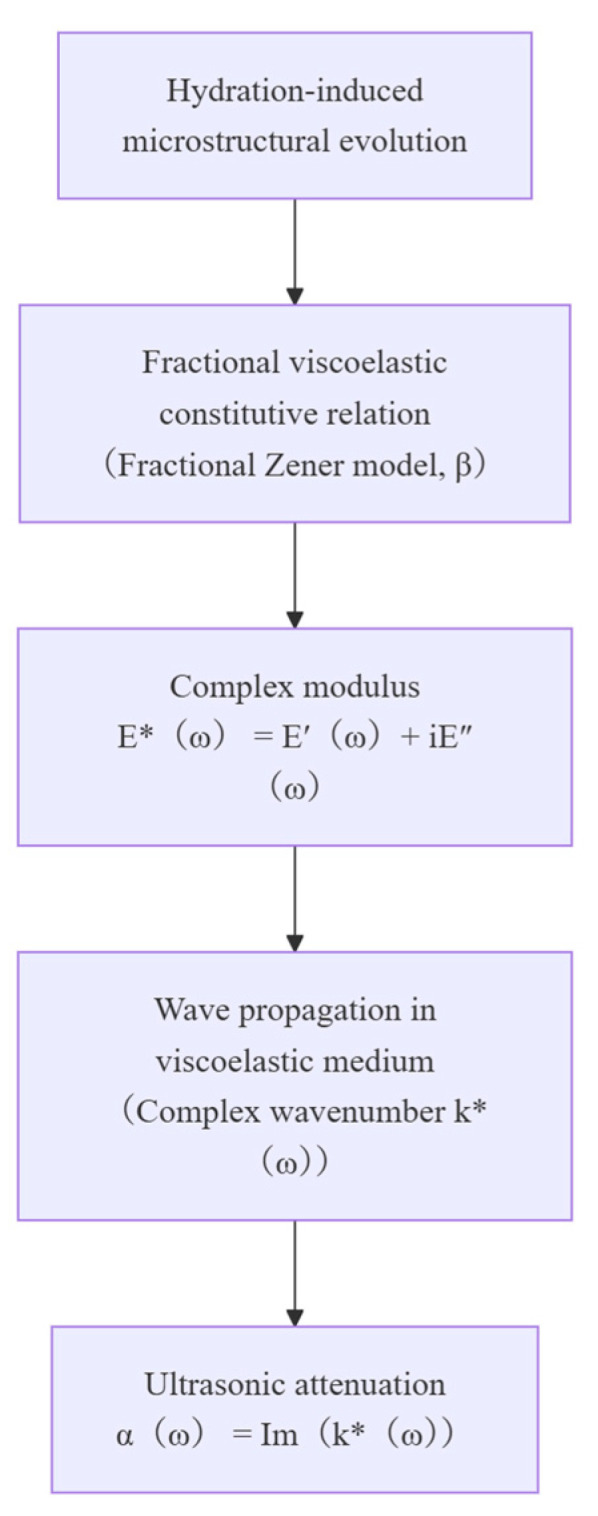
Flow diagram of the proposed fractional-order modeling framework, showing the transformation from fractional viscoelastic constitutive relations to the ultrasonic attenuation coefficient.

The overall modeling framework and the associated transformation from the fractional constitutive relation to the ultrasonic attenuation coefficient are schematically summarized in Figure 2.

**Figure 3:**
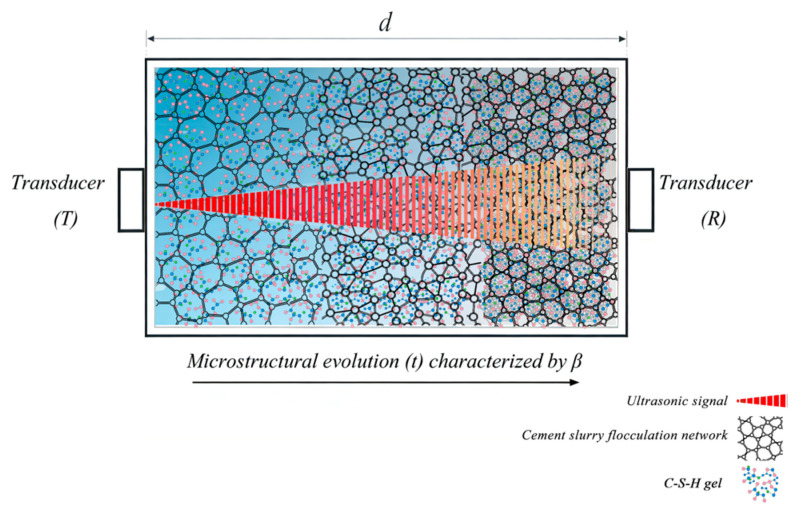
Schematic diagram of ultrasonic wave propagation in cement paste with flocculated structure evolution.

To establish a direct connection between the fractional constitutive model and ultrasonic testing, it is essential to define the propagation scenario of acoustic waves in cement paste as a viscoelastic medium. As shown in Figure 3, the temporal evolution of the flocculated network during resting—characterized by the fractional order (β)—is a key factor influencing ultrasonic propagation. A plane wave generated by the transmitting transducer (T) undergoes attenuation while traveling through this evolving medium because its energy is scattered by the flocculated structures. This leads to a reduced signal amplitude at the receiving transducer (R), and such attenuation can be quantified by the attenuation coefficient (α(ω)). The following wave equation is formulated and solved to theoretically describe this physical process.

Assuming the material is unbounded and homogeneous, boundary effects are neglected. Although practical cement paste exhibits particle-level heterogeneity, it can be regarded as macroscopically homogeneous. In such an unbounded homogeneous viscoelastic medium, consider a plane ultrasonic wave propagating along the (x)-direction. Its dynamics are governed by the classical wave equation, whose frequency-domain form is given by:

(7) $$-\rho \omega^{2}u(\omega )=\frac{\partial \sigma }{\partial x}$$

Here, ρ is the density of the paste, and u(ω) is the displacement field in the frequency domain. The wave equation simultaneously involves the stress σ and the displacement u, which must be coupled through the constitutive relation of the medium. The infinitesimal strain ε(ω) induced by the acoustic wave is defined by the displacement gradient as:

(8) $$\varepsilon (\omega )=\frac{\partial u(\omega )}{\partial x}=iku(\omega )$$

Combining the constitutive relation σ(ω)=E∗(ω)ε(ω) with the strain definition (8), the stress expression can be obtained as:

(9) $$\sigma (\omega )=E^{*}(\omega )\cdot iku(\omega )$$

Taking the spatial derivative of Equation (9) and substituting it into the wave Equation (7) yields:

(10) $$\frac{\partial \sigma }{\partial x}=ik\cdot E^{*}(\omega )\cdot iku(\omega )=-k^{2}E^{*}(\omega )u(\omega )$$

Combining Equations (7) and (10) and eliminating u(ω), the expression for the complex wavenumber (k) is obtained as:

(11) $$-\rho \omega^{2}u(\omega )=-k^{2}E^{*}(\omega )u(\omega )\Rightarrow k^{2}=\frac{\rho \omega^{2}}{E^{*}(\omega )}$$

Therefore,

(12) $$k=\omega \sqrt{\frac{\rho }{E^{*}(\omega )}}$$

Since $E^{*}(\omega )$ is complex, k is also complex, and its imaginary part corresponds to the ultrasonic attenuation coefficient α(ω), i.e., $k=k^{\prime }+i\alpha (\omega )$.

To obtain an explicit solution, the numerator and denominator in Equation (5) are rewritten in polar form. Let the denominator be:

(13) $$1+{\left(i\omega \tau \right)}^{\beta }=1+{\left(\omega \tau \right)}^{\beta }e^{i\beta \pi /2}=R_{d}e^{i\phi_{d}}$$

where

(14) $$R_{d}=\sqrt{1+2{\left(\omega \tau \right)}^{\beta }\cos(\beta \pi /2)+{\left(\omega \tau \right)}^{2\beta }}$$

(15) $$\phi_{d}=\arctan\left(\frac{{\left(\omega \tau \right)}^{\beta }\sin(\beta \pi /2)}{1+{\left(\omega \tau \right)}^{\beta }\cos(\beta \pi /2)}\right)$$

Let the numerator be:

(16) $$E_{\infty }+E_{0}{\left(i\omega \tau \right)}^{\beta }=E_{\infty }+E_{0}{\left(\omega \tau \right)}^{\beta }e^{i\beta \pi /2}=Me^{i\phi_{m}}$$

where,

(17) $$M=\sqrt{E_{\infty }^{2}+E_{0}^{2}{\left(\omega \tau \right)}^{2\beta }+2E_{0}E_{\infty }{\left(\omega \tau \right)}^{\beta }\cos(\beta \pi /2)}$$

(18) $$\phi_{m}=\arctan\left(\frac{E_{0}{\left(\omega \tau \right)}^{\beta }\sin(\beta \pi /2)}{E_{\infty }+E_{0}{\left(\omega \tau \right)}^{\beta }\cos(\beta \pi /2)}\right)$$

Substituting the polar forms into Equation (5), the complex modulus can be simplified as:

(19) $$E^{*}(\omega )=\frac{Me^{i\phi_{m}}}{R_{d}e^{i\phi_{d}}}=\frac{M}{R_{d}}e^{i(\phi_{m}-\phi_{d})}$$

Substituting Equation (19) into Equation (12) and using the square-root operation rule for complex numbers:

(20) $$k=\omega \sqrt{\frac{\rho }{E^{*}(\omega )}}=\omega \sqrt{\rho }\cdot \sqrt{\frac{R_{d}}{M}e^{-i(\phi_{m}-\phi_{d})}}=\omega \sqrt{\frac{\rho R_{d}}{M}}e^{-i(\phi_{m}-\phi_{d})/2}$$

Therefore, the attenuation coefficient α(ω) is the imaginary part of the complex wavenumber k:

(21) α(ω)=Im(k)=ωρRdMsinϕd−ϕm2

Equation (21) contains the phase difference. To obtain a more intuitive physical interpretation, it is necessary to simplify it. According to the trigonometric identity $\sin((\phi_{d}-\phi_{m})/2)$, an explicit result can be obtained via the sine difference formula. Let $A={\left(\omega \tau \right)}^{\beta }\sin(\beta \pi /2)$, $B_{d}=1+{\left(\omega \tau \right)}^{\beta }\cos(\beta \pi /2)$, and $B_{m}=E_{\infty }+E_{0}{\left(\omega \tau \right)}^{\beta }\cos(\beta \pi /2)$, then $\tan\phi_{d}=A/B_{d}$ and $\tan\phi_{m}=E_{0}A/B_{m}$. Using the trigonometric formulas for the sum and half of angles, Equation (21) can be shown to be equivalent to the following form:

(22) α(ω)=Im(k)=ω2c∞⋅(E0−E∞)(ωτ)βsin(βπ/2)E∞2+E02(ωτ)2β+2E0E∞(ωτ)βcos(βπ/2)

where $c_{\infty }=\sqrt{E_{\infty }/\rho }$ is the acoustic velocity. This equation quantitatively characterizes the coupling relationship among acoustic attenuation, frequency ω, and the fractional order β.

A smaller β leads to pronounced velocity dispersion, while as β→1, the system approaches the elastic limit and the acoustic velocity becomes nearly independent of frequency.

**Figure 4:**
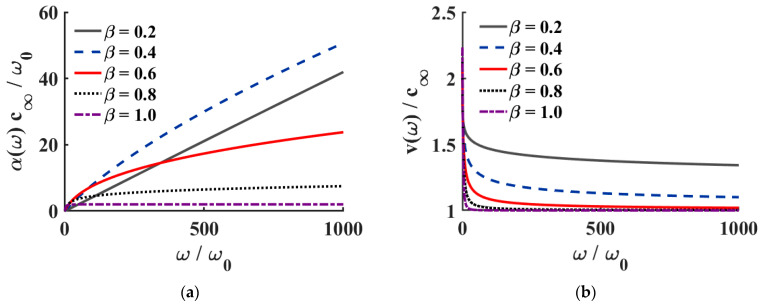
Ultrasonic propagation characteristics of the fractional viscoelastic model in the frequency domain. (**a**) Variation in the normalized ultrasonic attenuation coefficient with normalized frequency. (**b**) Variation in the normalized phase velocity with normalized frequency.

In the fractional Zener model, the viscoelastic properties of the medium influence not only ultrasonic attenuation but also velocity dispersion. Figure 4a presents the variation in the normalized ultrasonic attenuation coefficient $\alpha (\omega )c_{\infty }/\omega_{0}$ with normalized frequency $\omega /\omega_{0}$ for different fractional orders β. When β is small, the attenuation coefficient increases significantly with frequency, indicating strong energy scattering within the medium. As β increases, the growth rate of attenuation is markedly reduced, and the medium gradually transitions toward a low-loss elastic state. This trend agrees with the theoretical prediction of Equation (22), demonstrating that the fractional parameter β plays a key role in governing band-limited attenuation behavior.

Figure 4b shows the variation in the corresponding normalized phase velocity $v(\omega )/c_{\infty }$ with frequency. The results indicate that the phase velocity is relatively low in the low-frequency region and gradually increases with frequency, eventually reaching a plateau at high frequencies. When β is small, the medium exhibits pronounced dispersion; as β increases, the dispersion progressively weakens, and in the limit β→1 the medium approaches an ideal elastic solid, for which the wave velocity is nearly independent of frequency. This phenomenon confirms that the fractional Zener model can accurately describe the velocity dispersion induced by band-limited attenuation, providing a theoretical basis for interpreting subsequent experimental results and for parameter inversion.

### 2.4. Model Degeneration Verification

Based on the fractional constitutive relation, this subsection derives the ultrasonic wave attenuation in a viscoelastic cementitious medium. The key idea is that material viscoelasticity introduces frequency-dependent energy dissipation, which manifests as a complex modulus and, consequently, a complex wavenumber. The imaginary part of the wavenumber directly governs the measurable ultrasonic attenuation coefficient.

Under the assumptions stated in Section 2.3, the ultrasonic wave propagation and attenuation are derived as follows.

To verify the correctness and general applicability of the proposed model, the model is reduced to two classical limiting cases: a viscous fluid (β→0) and an elastic solid (β→1). This demonstrates that the model is consistent with classical theory under limiting conditions.

In the viscous-fluid limit (β→0), the equilibrium modulus $E_{\infty }$ of the material is much smaller than the instantaneous modulus $E_{0}$, and the mechanical response is dominated by viscosity. To further degenerate the model into a purely viscous fluid (Newtonian fluid), one additional step is needed: letting $E_{\infty }\to 0$. Through small-parameter asymptotic analysis, one can show that the attenuation coefficient α(ω) is proportional to $\omega^{2}$, which corresponds to the classical acoustic attenuation law of Newtonian fluids.

Considering Equation (22), the asymptotic behavior as β→0 is analyzed as follows: ${\left(\omega \tau \right)}^{\beta }=e^{\beta \ln(\omega \tau )}\approx 1+\beta \ln(\omega \tau )$ (retaining the first-order term of the Taylor expansion), sin(βπ/2)≈βπ/2 (small-angle approximation), cos(βπ/2)≈1 (small-angle approximation), Substituting these into the numerator gives: $\left(E_{0}-E_{\infty }){\left(\omega \tau \right)}^{\beta }\sin(\beta \pi /2)\approx (E_{0}-E_{\infty })\cdot (1+\beta \ln(\omega \tau ))\cdot (\beta \pi /2\right)$ retaining terms up to first order in β:

(23) $$numerator\approx (E_{0}-E_{\infty })\cdot \frac{\beta \pi }{2}$$

Substituting into the square-root term in the denominator:

(24) $$\begin{array}{l} E_{\infty }^{2}+E_{0}^{2}{\left(\omega \tau \right)}^{2\beta }+2E_{0}E_{\infty }{\left(\omega \tau \right)}^{\beta }\cos(\beta \pi /2)\\ \approx E_{\infty }^{2}+E_{0}^{2}(1+2\beta \ln(\omega \tau ))+2E_{0}E_{\infty }(1+\beta \ln(\omega \tau )) \end{array}$$

Combining the constant term and the β-dependent term yields:

(25) $$denominator\approx (E_{\infty }+E_{0})\left[1+\frac{\beta (E_{0}^{2}+E_{0}E_{\infty })\ln(\omega \tau )}{{\left(E_{\infty }+E_{0}\right)}^{2}}\right]$$

Combining the numerator and denominator yields:

(26) $$\alpha (\omega )\approx \frac{\omega }{2c_{\infty }}\cdot \frac{(E_{0}-E_{\infty })\frac{\beta \pi }{2}}{(E_{\infty }+E_{0})\left[1+\frac{\beta (E_{0}^{2}+E_{0}E_{\infty })\ln(\omega \tau )}{{\left(E_{\infty }+E_{0}\right)}^{2}}\right]}$$

When β→0, the β-dependent terms in the denominator can be neglected:

(27) $$\alpha (\omega )\approx \frac{\omega }{2c_{\infty }}\cdot \frac{(E_{0}-E_{\infty })\beta \pi }{2(E_{\infty }+E_{0})}$$

In the viscous-fluid limit, the material response is predominantly viscous and elasticity can be neglected, which requires $E_{\infty }\to 0$ (the long-term modulus approaches zero). Meanwhile, the fractional parameter β is linked to the viscosity η through the relaxation time τ $\tau ={\left(\eta /E_{0}\right)}^{1/\beta }$. As β→0, $\beta \ln(\omega \tau )=\beta \ln\omega +\ln(\eta /E_{0})$ must remain finite, implying that $\beta \ln(\eta /E_{0})$ tends to a constant. A more rigorous derivation shows that, under the double limit β→0 and $E_{\infty }\to 0$, we have:

(28) $$\alpha (\omega )\approx \frac{\omega^{2}\eta }{2\rho c^{3}}$$

Here, c denotes the acoustic velocity; in a purely viscous fluid, $c=\sqrt{E/\rho }$, where E is the effective modulus.

When β→1, the material behaves as an elastic solid. We directly substitute β=1 and consider the high-frequency approximation to demonstrate that the attenuation coefficient α(ω) is proportional to ω, which is consistent with the acoustic attenuation characteristics of elastic solids.

Substituting the exact value β=1 into Equation (22) and simplifying yields:

(29) $$\alpha (\omega )=\frac{\omega }{2c_{\infty }}\cdot \frac{(E_{0}-E_{\infty })\omega \tau }{\sqrt{E_{\infty }^{2}+E_{0}^{2}{\left(\omega \tau \right)}^{2}}}$$

In the elastic-solid limit (β→1), the material response is dominated by elasticity and viscous effects become negligible. This corresponds to the high-frequency limit (ωτ≫1) or ($E_{0}\gg E_{\infty }$). When $E_{0}^{2}{\left(\omega \tau \right)}^{2}\gg E_{\infty }^{2}$, we have $\sqrt{E_{\infty }^{2}+E_{0}^{2}{\left(\omega \tau \right)}^{2}}\approx E_{0}\omega \tau$. Substituting this approximation yields:

(30) $$\alpha (\omega )\approx \frac{\omega }{2c_{\infty }}\cdot \frac{(E_{0}-E_{\infty })\omega \tau }{E_{0}\omega \tau }=\frac{\omega }{2c_{\infty }}\cdot \frac{E_{0}-E_{\infty }}{E_{0}}$$

Since $c_{\infty }=\sqrt{E_{\infty }/\rho }$, substituting this relation yields the further simplified form:

(31) $$\alpha (\omega )\approx \frac{\omega }{2\sqrt{E_{\infty }/\rho }}\cdot \frac{E_{0}-E_{\infty }}{E_{0}}=\frac{\omega \sqrt{\rho }}{2\sqrt{E_{\infty }}}\cdot \frac{E_{0}-E_{\infty }}{E_{0}}$$

To remain consistent with the classical form, we note that in an elastic solid the acoustic velocity is $c=\sqrt{E/\rho }$, and $c^{3}={\left(E/\rho \right)}^{3/2}=E^{3/2}/\rho^{3/2}$ therefore:

(32) $$\frac{1}{\rho c^{3}}=\frac{1}{\rho \cdot (E^{3/2}/\rho^{3/2})}=\frac{\sqrt{\rho }}{E^{3/2}}$$

Therefore, the above expression can be written as:

(33) $$\alpha (\omega )=\omega \cdot \frac{\left|E_{0}-E_{\infty }\right|}{2\rho c^{3}E_{\infty }}$$

Here, c adopts the long-term acoustic velocity $c_{\infty }$.

The proposed fractional model correctly degenerates to classical theories under both limiting conditions. When β→0, small-parameter expansion and double-limit analysis demonstrate that α(ω) is proportional to $\omega^{2}$, consistent with the acoustic attenuation law of Newtonian fluids. When β→1, high-frequency approximation shows that α(ω) is proportional to ω, matching the attenuation behavior of Hookean elastic solids.

In summary, the limiting cases β→0 and β→1 recover the classical viscous-dominated and elastic-dominated responses, respectively, confirming the physical consistency of the proposed formulation. These results demonstrate that the fractional model provides a continuous and unified description bridging the two extremes, while the detailed derivations are retained above for completeness.

## 3. Materials and Methods

This study employs fractional rheological theory to quantitatively characterize the microstructural evolution of cement-based materials during the setting process using ultrasonic monitoring. The experimental section systematically details the material preparation, ultrasonic testing system, experimental procedures, and data processing methods, ensuring reproducibility and precise control throughout the testing process.

### 3.1. Material Preparation and Specimen Design

The experiments employed P·O 42.5 ordinary Portland cement (Jiuchang Building Materials Business Department, Shandong, China), whose chemical composition is shown in Table 1 and complies with the GB/T 176-2017 standard [29]. A polycarboxylate-based superplasticizer (Dongrun Baisheng New Materials Co., Ltd., Sichuan, China) (PCE) was added at 0.2% of the cement mass. In addition to the chemical composition summarized in Table 1, the cement paste density and ultrasonic wave speed were measured at the initial stage to provide baseline material properties for subsequent analysis. The initial bulk density of the fresh cement paste was approximately 1900 kg/m^3^, and the corresponding longitudinal ultrasonic wave velocity was on the order of (1.2–1.8) × 10^3^ m/s, which is consistent with typical values reported for early-age cement pastes. These properties primarily affect the absolute magnitude of wave propagation parameters but do not alter the trend-based identification of the fractional parameter β during hydration.

To investigate the influence of the water–cement ratio on rheological behavior, four levels were selected: 0.40, 0.45, 0.50, and 0.55. For each mixture, 500 mL of cement paste was prepared. The specific mix proportions were as follows: cement was fixed at 1000 g, and the corresponding water masses were 400 g, 450 g, 500 g, and 550 g, respectively. The PCE dosage was uniformly maintained at 2.0 g.

Mixing was carried out in a controlled-temperature environment (25 ± 1 °C). A planetary mixer was used to dry-mix cement and superplasticizer at 140 r/min for 1 min, after which water was added and wet-mixing was performed at 285 r/min for 3 min to ensure uniform dispersion without agglomeration. The fresh paste was poured into a custom acrylic container (150 mm × 50 mm × 50 mm), allowed to rest for 5 min to eliminate entrapped air bubbles, and then subjected to the monitoring stage.

### 3.2. Design of the Ultrasonic Monitoring System

The ultrasonic monitoring system was constructed based on a through-transmission configuration. The core transmitting and receiving components were a pair of 25 kHz piezoelectric ceramic transducers (model: DYW-H25-200CTY) (Dayu Electronics Technology Co., Ltd., Fujian, China), with a center frequency of 25 kHz ± 1 kHz. The transducers exhibit a broadband frequency response covering the excitation frequency range used in this study, ensuring stable signal transmission and reception during early-age monitoring. The choice of low-frequency PZT elements minimizes scattering effects and enhances penetration in highly attenuative fresh cement paste. The transducers were mounted on opposite sides of an acrylic container, with a fixed sensing distance of 150 mm.

The excitation signal was generated by an STM32F407 microcontroller (STMicroelectronics, Geneva, Switzerland) and shaped using a Hanning-windowed 5-cycle sinusoidal burst. After amplification by a high-voltage driver, a 50-Vpp excitation pulse was delivered to the transmitting transducer. The received signal first passed through a 40 dB preamplifier and was then sampled by a 16-bit ADC module at a sampling rate of 1 MHz.

To compensate for temperature-induced variations in acoustic velocity, the system incorporated an NTC thermistor connected via an SPI interface to monitor the ambient temperature (accuracy ±0.5 °C). The measured temperature was used to correct sound velocity according to the empirical relation $c(T)=c_{0}+0.6(T-25)$ where $c_{0}=1480\;m/s$. Temperature effects were controlled by conducting all experiments in a temperature-stabilized laboratory environment. In addition, temperature was continuously monitored during the test period to ensure that fluctuations remained within a narrow range. The proposed framework relies on baseline-referenced attenuation trends rather than absolute signal amplitudes, which inherently reduces sensitivity to minor temperature-induced variations. The effectiveness of temperature compensation was verified by comparing attenuation trends under similar hydration conditions, confirming consistent evolution of the identified fractional parameter β.

### 3.3. Experimental Protocol and Procedure

**Figure 5:**
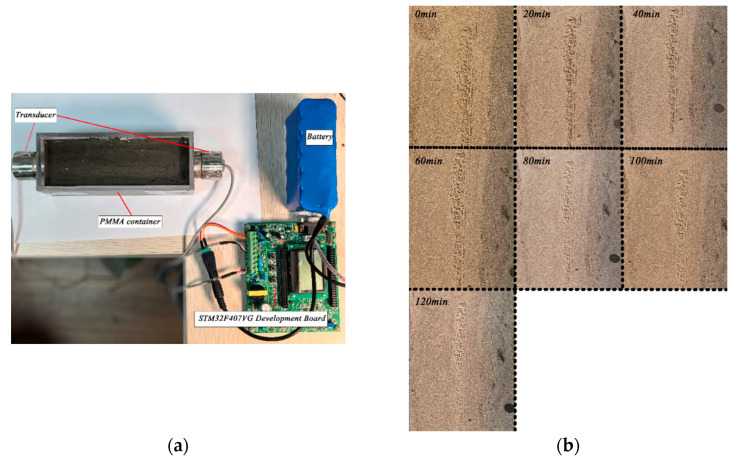
Experimental setup and early-age evolution of cement paste (0–120 min). (**a**) Experimental setup, (**b**) Apparent morphology evolution of cement paste from 0 to 120 min.

Each experiment lasted 120 min and began immediately after paste preparation and the subsequent resting period. Ultrasonic signals were collected every 5 min. At the initial stage, the system was calibrated using deionized water as the reference medium. During the experiments, the ambient temperature was maintained at 25 ± 1 °C and the relative humidity at 50 ± 5% RH. The baseline amplitude $A_{0}$ and temperature $T_{0}$ were recorded throughout the process. Figure 5 experimental setup for embedded ultrasonic sensing and the early-age evolution of cement paste observed from 0 to 120 min.

During formal measurements, three pulse signals were acquired in each cycle and averaged to suppress random noise. At the end of each test, all time-domain signals and temperature data were stored for subsequent analysis. To ensure data consistency, each water–cement ratio level was tested in triplicate, and the final results were obtained by averaging after removing outliers.

### 3.4. Data Processing and Parameter Inversion

Ultrasonic signal processing was performed using a time-domain attenuation approach. The raw signals were first corrected for DC offset to eliminate hardware-induced baseline drift. Subsequently, a Hilbert transform was applied to extract the amplitude envelope of the signal, representing the acoustic energy decay. To suppress noise interference, the envelope data were smoothed using a moving-average filter.

Based on the physical model of amplitude decay in a viscoelastic medium ($A(x)=A_{0}e^{-\alpha x}$), the natural logarithm of the envelope amplitude within the steady-attenuation region was taken and linearly fitted against propagation distance. To extract the ultrasonic attenuation coefficient, the received signal was first processed using an envelope detection method. The attenuation region was selected from the main transmitted wave packet after excluding the initial excitation transient and late-time reflections from specimen boundaries. Specifically, a time window corresponding to the stable propagation region with high signal-to-noise ratio was identified, and the exponential decay of the envelope within this region was fitted to determine the attenuation coefficient. This procedure ensures consistency across repeated measurements and minimizes the influence of noise and boundary effects. The slope of the fitted line corresponds to the decay rate γ (unit: s^−1^), and the attenuation coefficient α (unit: Np/m) was obtained using α=γ/c where c is the sound velocity.

The density ρ of each sample was measured immediately after specimen preparation and used as a constant input parameter. The instantaneous elastic modulus $E_{0}$ was calculated from the propagation theory of elastic waves in solids, using the measured sound velocity c and density ρ.

The long-term elastic modulus $E_{\infty }$ was estimated using the effective modulus method, whose theoretical basis originates from effective medium theory in cementitious materials. This method simplifies long-term creep and viscoelastic effects into a reduced elastic modulus, generally expressed as $E_{\infty }=E_{0}/(1+\varphi_{\infty })$ where $\varphi_{\infty }$ is the ultimate creep coefficient.

Considering the early-age characteristics of cementitious materials in this study, $\varphi_{\infty }$ was parameterized using an empirical relation associated with the water–cement ratio: $\varphi_{\infty }=k\cdot {\left(w/c/0.5\right)}^{2}$ which enables rapid estimation and reflects the influence of mix proportion on flow and deformation behavior.

Finally, the coefficient of determination ($R^{2}$) was calculated to evaluate the goodness of linear fits, ensuring the reliability of parameter inversion.

Each experiment was repeated three times under identical conditions to assess repeatability. Outliers were screened by comparing signal quality and attenuation trends; no abnormal measurements inconsistent with physical expectations were observed. For each time point, the mean attenuation coefficient is reported, while the experimental variability is represented by the minimum and maximum values among the three repeated measurements. This approach emphasizes repeatability and trend consistency rather than statistical significance testing, which is appropriate for early-age monitoring studies.

## 4. Results and Discussion

The core objective of this section is to integrate experimental data with the theoretical model to systematically analyze time-domain signal characteristics, ultrasonic attenuation behavior, variations in the fractional order β, and multi-model comparisons. These analyses collectively reveal the evolution of the microstructure as it transitions from a viscous fluid to an elastic solid. Cement pastes with different water–cement ratios (Section 3.1) were tested using the ultrasonic monitoring system, and the results were compared with the theoretical predictions of the fractional constitutive model (Equation (5)), providing a quantitative basis for monitoring the rheological properties of cement-based materials.

### 4.1. Evolution Characteristics of Typical Time-Domain Signals of Cement Paste

**Figure 6:**
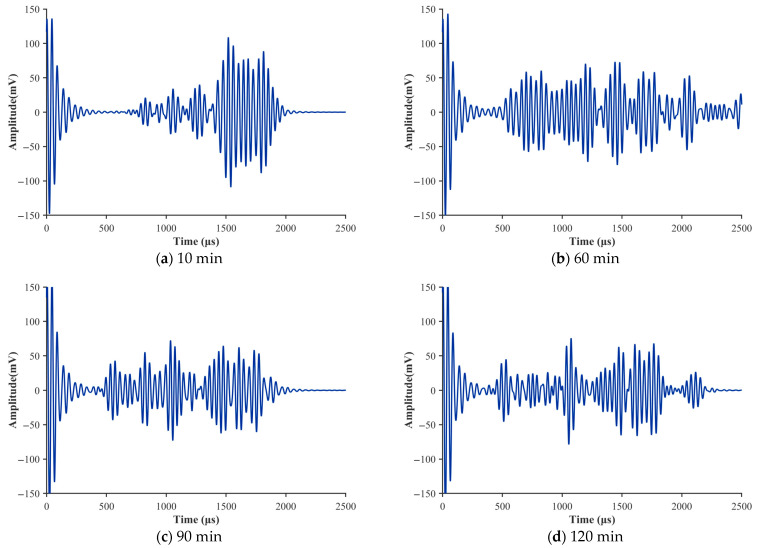
Time-domain signals of cement paste (W/C = 0.4) at different hydration times.

As shown in Figure 6, taking the ultrasonic signal of cement paste with a water–cement ratio of 0.40 as an example, the time-domain responses at four key moments—10 min, 60 min, 90 min, and 120 min—were comparatively analyzed. At 10 min (a), the signal exhibits characteristics typical of a viscous-fluid-dominated state. The overall amplitude remains relatively high, but the envelope decays extremely rapidly, with oscillations vanishing within a very short duration and the waveform appearing irregular. This indicates that, during the early stage of setting, the dispersed cement particles are separated by free water, and the pronounced viscous dissipation causes ultrasonic energy to be quickly attenuated during propagation. At this moment, the material possesses very low structural strength, and its mechanical behavior closely resembles that of a Newtonian fluid. In terms of the fractional Zener model, this corresponds to a fractional order β approaching zero, meaning that the constitutive response is dominated by viscous behavior.

As hydration progresses to 60 min (b) and 90 min (c), the time-domain signals exhibit clear transitional characteristics. Compared with the 10 min response, the most notable change is the increased persistence of oscillations. Beyond 500 μs, multiple cycles of regular, continuous high-frequency oscillations become visible. Although the peak amplitude decreases, the envelope attenuation rate is substantially reduced.

This transformation signifies fundamental microstructural evolution within the paste: hydration products (C–S–H gels) form progressively and interconnect to establish an initial three-dimensional flocculated network. This network enhances the continuity of the medium and strengthens its elastic response. Consequently, the ultrasonic energy-loss mechanism shifts from purely viscous dissipation toward viscoelastic damping, with elastic wave-propagation characteristics becoming increasingly pronounced.

In terms of fractional-order theory, this evolution is reflected by the gradual increase in the fractional order β toward 1, indicating that the material’s “memory” and elastic contribution are continuously strengthened, and the constitutive behavior transitions from a fractional derivative toward an integer-order form.

By 120 min (d), the time-domain signal has evolved to exhibit propagation characteristics dominated by an elastic solid. The signal amplitude stabilizes at a relatively low level, and the waveform displays highly regular, continuous high-frequency oscillations, with only minimal attenuation throughout the entire observation window. This indicates that a dense and rigid spatial network structure has formed within the paste, where particles are interconnected through strong bonding forces.

In such a medium, ultrasonic energy loss arises primarily from elastic scattering caused by microstructural inhomogeneities (e.g., unhydrated particles, microvoids) rather than viscous friction. This stage corresponds to a further increase in the fractional order β in the fractional model, reflecting the strengthening of elastic behavior and the near-solid-like mechanical response of the material.

### 4.2. Verification of the Fractional Model and Analysis of Attenuation Characteristics

**Figure 7:**
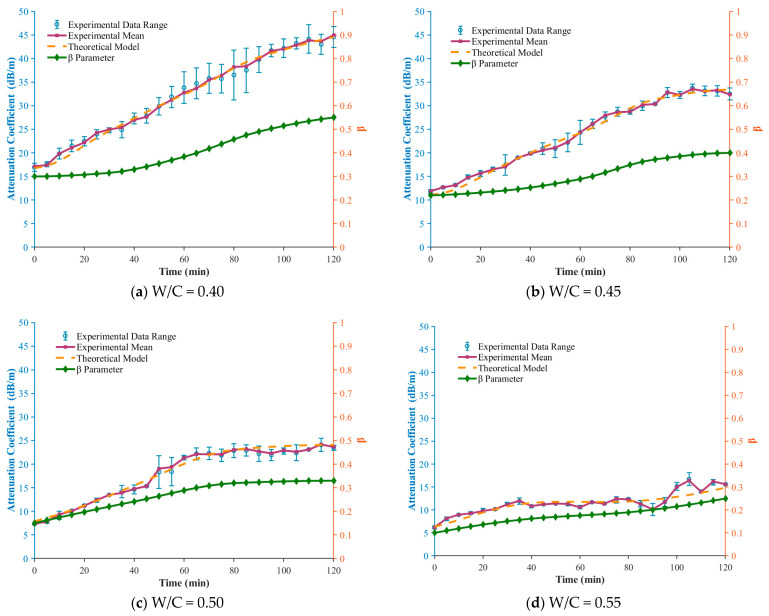
Ultrasonic attenuation coefficients of cement pastes with different water–cement ratios. Error bars indicate the experimental range, with upper and lower bounds corresponding to the maximum and minimum values obtained from three independent measurements (n = 3).

To validate the effectiveness of the proposed fractional Zener model in describing ultrasonic attenuation during the setting of cement paste, this section systematically compares experimental measurements with model predictions for water–cement ratios (W/C) of 0.40, 0.45, 0.50, and 0.55. Figure 7 illustrate the evolution of the attenuation coefficient α over time (0–120 min) for each water–cement ratio. Each figure presents the experimental mean value, the fluctuation range of the measurements, the theoretical prediction curve, and the corresponding evolution of the fractional order β, which serves as a key parameter in the model.

As shown in the figures, for all water–cement ratios, the experimentally measured attenuation coefficients α (blue circles) exhibit a variation trend that is highly consistent with the theoretical predictions based on the fractional model (Equation (22), red squares). Both curves increase monotonically with setting time, which corroborates the earlier time-domain signal analysis: as hydration products accumulate and the flocculated network develops, the dominant attenuation mechanism transitions from early-stage viscous dissipation to later-stage elastic scattering, resulting in a continuous increase in the macroscopic attenuation coefficient α.

To quantitatively assess the prediction accuracy of the model, the relative error (RE) between the theoretical predictions and the experimental mean values was calculated over the entire setting process. The results show that the model performs well across all water–cement ratios, although the prediction accuracy varies:

1. W/C = 0.40 (a): The average relative error is 1.85%, with the maximum error (~7.96%) occurring at the early stage. This indicates that for denser pastes with lower water–cement ratios, the fractional model can capture the attenuation characteristics with very high precision.
2. W/C = 0.45 (b): The average relative error is 3.33%. The strong agreement between the predicted curve and the experimental mean further confirms the robustness of the model.
3. W/C = 0.50 (c): The average relative error is 3.35%. Although the fluctuation range of the experimental data (grey region) is larger—suggesting slightly poorer microstructural uniformity at this water–cement ratio—the model still effectively tracks the overall trend.
4. W/C = 0.55 (d): The average relative error is 7.11%, the highest among all groups. This suggests that at higher water–cement ratios, the greater amount of free water leads to more complex rheological behavior, introducing certain limitations to the model’s predictive capability.

As the key parameter of the proposed model, the fractional order β provides a unique perspective on the microstructural evolution of cement paste. The temporal variation curves (green dash–dot lines) in all four figures exhibit a consistent trend: β starts from a relatively low value (0.1–0.3) in the early stage of setting and increases approximately linearly as hydration progresses, reaching the range of 0.33–0.55 at 120 min.

At the beginning of setting, β≈0 represents an ideal viscous-fluid response. In the experiments, the initial β values are greater than zero but far below one, indicating that although the fresh paste is dominated by viscous behavior, it already exhibits a certain degree of “memory” behavior—distinct from Newtonian fluids—which reflects the presence of an initial flocculated microstructure.

The monotonic increase of β directly captures the continuous formation and strengthening of the internal three-dimensional network. As hydration products such as C–S–H gel progressively interweave and interconnect, the solid-like (elastic) nature of the material increases, while the fluid-like (viscous) contribution diminishes. The inherent long-memory effect of fractional derivatives naturally describes this history-dependent microstructural reconstruction process.

Ultimately, the trend β→1 corresponds to the ideal Hookean elastic solid. Since β does not reach 1 by the end of the 120 min observation window, this indicates that hydration remains incomplete and the microstructure is still evolving, with the material exhibiting characteristics of a typical viscoelastic solid.

**Figure 8:**
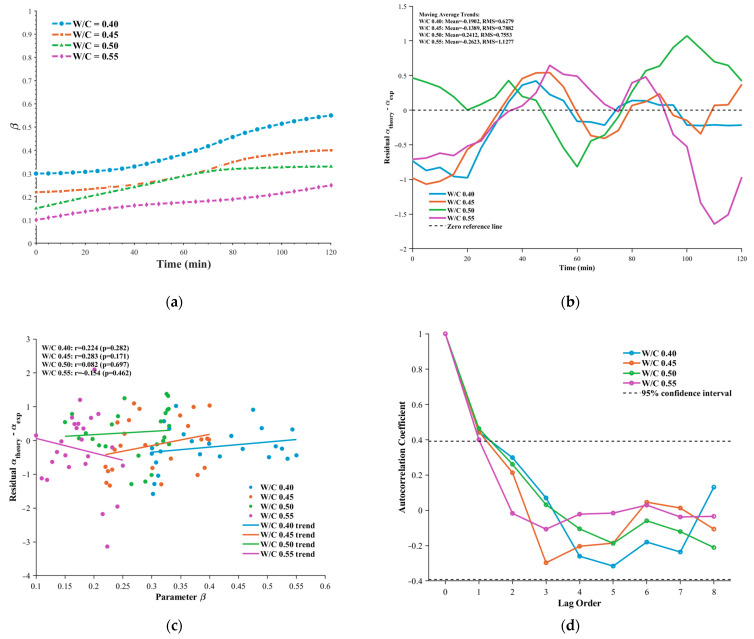
Comprehensive validation of the fractional-order model and residual analysis. (**a**) Time evolution of the fractional parameter β under different water–cement ratios, (**b**) Time series of residuals between model-predicted and experimental attenuation coefficients, (**c**) Correlation analysis between model residuals and the fractional parameter β (**d**) Autocorrelation analysis of the model residuals.

As shown in Figure 8a, for the same setting time, a higher water–cement ratio corresponds to a lower value of β. For example, at 120 min, β=0.55 for W/C = 0.40, whereas β=0.25 for W/C = 0.55. This quantitative relationship indicates that increasing the water–cement ratio dilutes the concentration of cement particles and slows down the formation rate of the flocculated network. Consequently, the paste exhibits stronger macroscopic fluidity (i.e., lower elasticity), which is fully consistent with traditional rheological understanding.

Figure 8b presents the temporal evolution of the residuals ($\alpha_{\mathrm{theory}}-\alpha_{\exp}$) between the model predictions and the experimental measurements. For all water–cement ratios, the mean residuals remain very close to zero (ranging from −0.26 to 0.24), indicating that the model predictions are essentially unbiased. In addition, the residual sequences fluctuate randomly around zero throughout the entire setting period, with no observable systematic trends, and the fluctuation magnitude is limited (root-mean-square error RMS between 0.63 and 1.13). These characteristics collectively demonstrate that the fractional model achieves high predictive accuracy for the attenuation coefficients of cement pastes across different water–cement ratios.

To further examine whether the model exhibits systematic bias, Figure 8c analyzes the relationship between the model residuals and the intrinsic parameter β. The scatter plots show that the residuals are randomly distributed around the zero line, and the trend lines for all water–cement ratios are nearly horizontal. Correlation analysis indicates that the absolute values of the correlation coefficients (r) are all below 0.3, and the corresponding (*p*)-values are much greater than the 0.05 significance threshold. This confirms that no statistically significant correlation exists between the residuals and the value of β. In other words, the prediction deviations of the model are random across different structural states (represented by different β values), rather than arising from any structural deficiency of the model itself.

Finally, autocorrelation analysis of the residual sequences (Figure 8d) shows that, for all cases, the autocorrelation coefficients decay and stabilize within the 95% confidence interval after lag 2. This indicates that the residuals exhibit no significant autocorrelation beyond the second lag and therefore satisfy the key characteristics of white noise. This result confirms that the fractional model has successfully captured the essential temporal dependencies in the evolution of the attenuation coefficient, with no important systematic time information left unmodeled.

Figure 6, Figure 7 and Figure 8 indicate that the evolution of the fractional parameter β is generally monotonic but not strictly linear over time. Instead, β exhibits stage-dependent trends that correspond well to the classical hydration process. During the dormant period, β increases slowly, reflecting a predominantly viscous response of the fresh cement paste. In the acceleration stage, a more pronounced increase in β is observed, associated with rapid formation of hydration products and the development of a load-bearing microstructure. As hydration progresses toward rigidity and early hardening, the growth rate of ββ gradually decreases, indicating a transition toward a more elastic-dominated viscoelastic state.

For mixtures with higher water–cement ratios, the prediction error of ultrasonic attenuation becomes more pronounced. This behavior can be attributed to increased variability associated with a higher free-water content, which enhances scattering, local heterogeneity, and transient coupling effects during early hydration. In such systems, the ultrasonic attenuation is more sensitive to small fluctuations in microstructure and pore water distribution, leading to increased dispersion in the measured signals. Consequently, deviations between predicted and measured attenuation are more noticeable at higher water–cement ratios, particularly at very early ages.

The sensitivity of β to measurement noise and algorithmic adjustments was examined by analyzing its temporal stability and repeatability across repeated measurements. Although small fluctuations in β may arise from signal noise or envelope fitting procedures, the identified β evolution is primarily governed by long-term trends rather than pointwise variations. Since β is extracted through baseline-referenced attenuation trends, its estimation is inherently less sensitive to random noise compared with instantaneous ultrasonic indicators. The observed smooth evolution of β across hydration stages suggests that the parameter predominantly reflects underlying physical dynamics rather than numerical artifacts.

In some cases, β approaches a quasi-stationary zone or plateau at more advanced hydration ages. This behavior does not indicate a loss of sensitivity but rather reflects the stabilization of the viscoelastic microstructure once the primary hydration reactions have largely completed. At this stage, the cement paste exhibits a relatively stable elastic-dominated response, and further changes in intrinsic energy dissipation mechanisms become less pronounced. Therefore, the plateau of β can be interpreted as a signature of early-age mechanical stabilization rather than a limitation of the sensing framework.

The practical use of α(t) and β(t) as hydration indicators is subject to several limitations. First, both parameters rely on baseline-referenced measurements and are therefore most effective during early-age hydration, when material evolution is monotonic and well controlled. Second, α(t) reflects cumulative attenuation effects and may be influenced by experimental conditions such as coupling and boundary effects, whereas β(t) is a model-derived parameter whose interpretation depends on the validity of the assumed viscoelastic framework. Consequently, α(t) and β(t) should be interpreted as complementary indicators of hydration progression rather than absolute material properties.

The proposed method exhibits limited sensitivity to random measurement noise, as both α(t) and β(t) are extracted from envelope-based attenuation trends and evaluated through temporal evolution rather than instantaneous values. Temperature fluctuations primarily affect absolute signal amplitudes and wave speeds; however, their influence on β(t) is mitigated by baseline referencing and trend-based interpretation. Transducer misalignment and coupling conditions may affect absolute attenuation levels, but their impact on the temporal evolution of β(t) is expected to be secondary, particularly when consistent sensor geometry and fixed excitation–reception paths are maintained.

While the present study focuses on early-age hydration, the proposed framework is, in principle, capable of detecting non-monotonic phenomena such as micro-cracking or segregation. Such processes are expected to introduce abrupt or irreversible changes in attenuation behavior and in the evolution of β(t), in contrast to the smooth and monotonic trends observed during hydration. However, distinguishing these mechanisms from environmental or operational effects would require auxiliary sensing information and extended validation, which is beyond the scope of the current work.

The current formulation and experimental validation are limited to cement paste and therefore represent a matrix-level framework. In fiber-reinforced or highly heterogeneous concrete, additional effects such as multi-scattering, anisotropy, and guided-wave propagation are expected to influence ultrasonic attenuation. While the fractional viscoelastic model remains applicable at the effective-medium level, its extension to such systems would require incorporating appropriate homogenization or multi-scattering models. This represents an important direction for future work.

The fractional-order framework is inherently frequency-dependent and can be naturally extended to other ultrasonic frequencies or multi-frequency excitation schemes. Since the fractional constitutive model describes viscoelastic behavior across a broad frequency range, β can be identified using different excitation frequencies, provided that the corresponding attenuation data are available. Multi-frequency measurements may further enhance parameter identifiability and robustness by constraining the inversion process and enabling frequency-domain consistency checks.

Taken together, the validation results for all four water–cement ratios demonstrate that the proposed fractional model not only provides high-accuracy predictions of ultrasonic attenuation during the setting of cement paste but also yields residuals that are genuinely random. This verifies the robustness and completeness of the model. Overall, the evolution of β provides a physically consistent and stage-sensitive indicator of hydration progression, offering a robust descriptor of early-age material state that complements conventional ultrasonic attenuation analysis.

### 4.3. Comparative Analysis Between the Fractional Model and Other Viscoelastic Theories

After validating the fractional model developed in this study, we compare it with three representative viscoelastic models: the DFL (Decoupled Fractional Laplacian) model, the Fractal Modification model, and the classical Kelvin–Voigt model [30,31]. All of these models aim to describe wave propagation in viscoelastic media, yet they differ substantially in theoretical foundations and application focus.

The proposed fractional model introduces the fractional derivative order β, which effectively captures the memory effects and gradual microstructural evolution occurring during the setting of cement paste. Its frequency-domain complex-modulus formulation provides a direct and intuitive representation of viscoelastic response.

The DFL model employs a decoupled fractional Laplacian operator and is particularly efficient for handling anisotropic attenuation in highly heterogeneous media. Its wavefield simulations allow clear separation of amplitude decay and phase dispersion.

The Fractal Modification model is derived from fractal theory, using parameters such as ${\omega \tau }_{V}$ or κR as scaling variables. It is especially suited for mineral slurries where particle irregularity and clustering are prominent.

In contrast, the Kelvin–Voigt model—an integer-order viscoelastic representation composed of a parallel spring–dashpot system—is simple and intuitive but cannot adequately capture frequency-independent attenuation or the dynamic microstructural evolution characteristic of cement paste during hydration.

**Figure 9:**
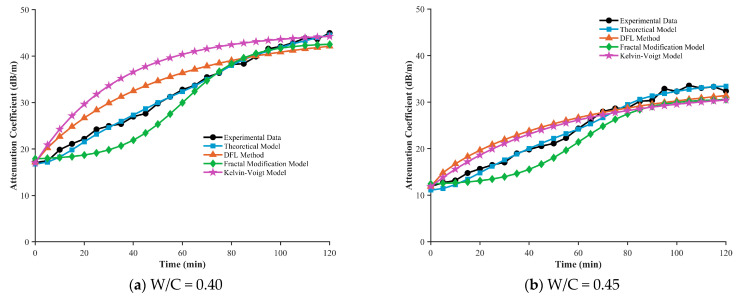

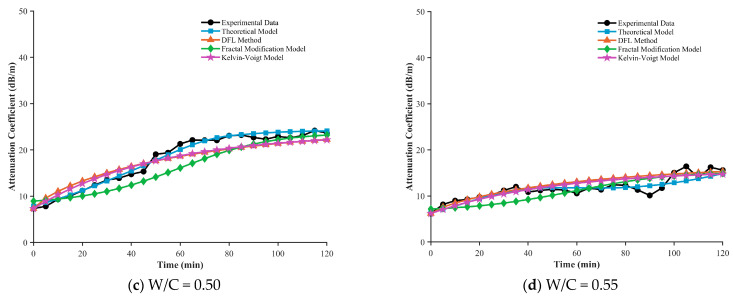
Comparison of model-predicted attenuation curves with experimental data of cement paste.

We performed a quantitative comparison between the proposed fractional model and three representative models—the DFL method, the Fractal Modification model, and the Kelvin–Voigt model—under different water–cement ratios (0.40, 0.45, 0.50, 0.55). The results are shown in Figure 9, and the statistical summary of average relative errors for each model is provided in the accompanying Table 2.

All models are able to capture the increasing trend of the attenuation coefficient over time, consistent with the physical evolution of the microstructure from a viscous fluid to a viscoelastic solid during cement hydration. However, the prediction accuracy varies markedly among the models. The fractional model proposed in this study exhibits the best performance across all water–cement ratios, with average relative errors of 1.85% (W/C = 0.40), 3.33% (W/C = 0.45), 3.54% (W/C = 0.50), and 7.11% (W/C = 0.55). It accurately reflects the temporal evolution of the attenuation coefficient.

In contrast, the prediction errors of the DFL method range from 10.61% to 12.32%, and those of the Fractal Modification model range from 8.07% to 13.16%, both significantly higher than those of the fractional model. As illustrated in (a) and (b), these models exhibit systematic overestimation in the early stage of setting (t < 60 min), particularly for W/C = 0.55, where the Fractal Modification model reaches an error of 13.16%. This behavior likely stems from the sensitivity of these models to the strong scattering effects induced by the rigid flocculated network formed at later hydration stages.

The classical Kelvin–Voigt model exhibits the highest instability among all models, with relative errors ranging from 9.27% to 18.42%. Notably, this model shows its largest error at a low water–cement ratio (W/C = 0.40, 18.42%), whereas the error becomes relatively smaller (9.27%) at a medium ratio (W/C = 0.50). This inconsistency reveals the difficulty of integer-order viscoelastic models in uniformly describing the complex viscoelastic behavior of cement pastes with different mix proportions.

Regarding the trend with water–cement ratio, prediction errors increase for all models as W/C increases from 0.40 to 0.55. For instance, the fractional model’s error increases from 1.85% to 7.11%, the DFL method from 10.61% to 10.71%, and the Fractal Modification model from 8.07% to 13.16%. This reflects that higher water–cement ratios introduce more free water and greater microstructural heterogeneity, thereby increasing the uncertainty in ultrasonic attenuation behavior. Despite this, the fractional model retains the highest prediction stability across all conditions: its maximum error (7.11%) is still lower than the minimum error of all other models, highlighting its advantage in modeling time-dependent material behavior through the fractional order β.

Overall, these results demonstrate that the fractional model driven by ultrasonic sensing can accurately capture the microstructural evolution during cement paste setting. The fractional parameter β serves as a real-time, sensitive indicator of the transition from viscous fluid to elastic solid. Compared with conventional rheometers—which require sample extraction and may disturb the hydration process—the proposed method enables continuous, non-invasive monitoring. Moreover, the model’s accurate description of low-frequency attenuation characteristics provides a solid theoretical basis for developing embedded real-time inversion algorithms.

From an SHM viewpoint, the monotonic and physically interpretable evolution of β observed in the experiments highlights its potential as a state variable rather than a purely empirical fitting parameter. During early hydration, β captures the transition from viscous-dominated to elastic-dominated behavior, while in later stages or under service conditions, changes in β may reflect alterations in the internal stress state, microcrack density, or damage-induced viscoelasticity. Compared with traditional ultrasonic velocity or amplitude indicators, β integrates attenuation behavior over a broad frequency band and is therefore less sensitive to measurement noise and boundary effects, which is advantageous for field-scale SHM applications.

The sensitivity of the proposed fractional-order ultrasonic model to aggregates, fibers, or reinforcement should be carefully discussed. In practical concrete, coarse aggregates introduce strong multiple scattering and impedance contrast, fibers induce anisotropic attenuation and dispersion, and steel reinforcement causes mode conversion and waveguiding effects. These factors primarily affect the propagation path and scattering characteristics of ultrasonic waves rather than the intrinsic viscoelastic behavior of the cementitious matrix.

From a modeling perspective, the fractional parameter β represents an effective viscoelastic state of the load-bearing matrix at the scale of ultrasonic wavelengths. In heterogeneous composites, β can therefore be interpreted as an equivalent or homogenized parameter that incorporates matrix behavior and mesoscale interactions. However, additional correction terms—such as aggregate-induced scattering losses or reinforcement-related boundary effects—would need to be explicitly incorporated into the attenuation model for quantitative interpretation in full concrete or reinforced concrete systems.

It should be acknowledged that the fractional parameter β is influenced by multiple physical factors and should not be interpreted as a hydration-exclusive variable in a strict sense. In the present study, β primarily reflects hydration-driven microstructural evolution because the experiments were conducted under well-controlled temperature and moisture conditions, with no external mechanical loading. Under such conditions, the dominant contribution to changes in β originates from the progressive formation of the cementitious network and the associated viscoelastic transition.

In practical SHM applications, however, additional environmental and operational factors may affect the inferred value of β. Temperature fluctuations can modify relaxation times and shift the frequency-dependent viscoelastic response, leading to apparent changes in β. Moisture gradients may alter pore-fluid distribution and local damping behavior, while mechanical loading or microcracking can introduce irreversible changes in energy dissipation mechanisms. These effects are expected to influence ultrasonic attenuation through different physical pathways compared with hydration-induced structural build-up.

From an identifiability perspective, β should therefore be regarded as an effective state parameter that integrates multiple contributions to viscoelastic attenuation. Decoupling hydration effects from environmental or damage-related influences requires auxiliary information and compensation strategies. For example, temperature effects can be mitigated through explicit temperature correction or multi-frequency normalization, while moisture-related variations may be constrained using humidity sensors or coupled poroelastic models. Load- or damage-induced changes in β are typically non-monotonic and irreversible, in contrast to the monotonic and smooth evolution observed during early-age hydration, providing a potential criterion for distinguishing different mechanisms.

Consequently, the practical interpretation of β in SHM should rely on baseline referencing and trend analysis rather than absolute values alone. When combined with environmental sensing, multi-parameter inversion, or digital twin frameworks, β can be effectively decoupled from external influences and used as a robust indicator of material state evolution and structural integrity.

Building upon the identifiability framework discussed above, the practical deployment of the proposed sensing approach depends on the robustness and stability of the ultrasonic measurement system under realistic conditions. While the laboratory system demonstrates reliable performance under controlled environments, its field applicability requires careful consideration of long-term sensor durability, signal stability, and installation-related variability. In cementitious environments, the durability of embedded piezoelectric (PZT) transducers is mainly governed by mechanical protection, chemical compatibility, and moisture ingress; previous SHM studies have shown that appropriate encapsulation or inert housing can ensure stable electromechanical performance even under alkaline and moisture-rich conditions.

From a signal interpretation perspective, the proposed framework relies on baseline-referenced attenuation trends rather than absolute signal amplitudes, which inherently enhances robustness against slow signal drift caused by temperature fluctuations, curing-induced stiffness evolution, or gradual coupling changes over days or weeks. Provided that a sufficient signal-to-noise ratio is maintained, the fractional parameter β can be consistently identified through trend analysis, supporting long-term monitoring applications. Although sensor coupling conditions and placement may affect absolute attenuation levels, their influence on the temporal evolution of β is expected to be secondary when consistent excitation–reception paths, standardized embedding procedures, and network-based redundancy are employed. Overall, these considerations indicate that the sensing system and interpretation strategy are compatible with in situ SHM applications beyond laboratory settings.

## 5. Conclusions

This study presented a physics-informed, fractional-order ultrasonic sensing framework for monitoring the early-age evolution of cementitious materials. By integrating a fractional Zener viscoelastic model with ultrasonic attenuation theory, a quantitative and physically interpretable relationship was established between hydration-driven microstructural changes and measurable acoustic attenuation. The fractional parameter β was shown to effectively characterize the continuous transition from viscous-dominated to elastic-dominated behavior during early hydration.

A custom ultrasonic measurement system, consisting of a low-frequency ultrasonic transducer, power amplifier, high-resolution data acquisition unit, and temperature compensation module, was developed to validate the proposed sensing approach. Experiments conducted on cement pastes with various water–cement ratios confirmed that the fractional-order model achieves significantly higher accuracy and robustness in capturing attenuation evolution compared with traditional integer-order and empirical models. The temporal evolution of β provides a clear, physically grounded indicator of structural build-up and agrees well with the known rheological and microstructural transitions of cement hydration.

It should be emphasized that, while this work concentrates on early-age cementitious materials, the proposed fractional-order ultrasonic framework is not limited to short-term hydration monitoring. On the contrary, early-age sensing establishes a physically consistent reference state that is essential for long-term SHM applications. By providing an interpretable viscoelastic baseline, the fractional parameter β enables the differentiation between intrinsic material evolution, environmental effects, and damage-induced anomalies during the service life of concrete structures. This capability is particularly valuable for intelligent construction, embedded sensing systems, and digital twin-based SHM, where material-scale information must be continuously linked to structural-scale performance assessment.

The proposed framework provides a promising foundation for next-generation, model-based ultrasonic sensing technologies aimed at quality assessment and structural state evaluation in high-end engineering applications, including large-scale concrete components, tunnel linings, marine foundations, and intelligent construction systems. By introducing a physically interpretable fractional parameter β, the framework establishes a direct link between microstructural evolution and measurable ultrasonic attenuation, offering a compact and robust sensing strategy that is well suited for integration into sensor-empowered intelligent construction and digital twin frameworks for cement-based infrastructure.

Nevertheless, the present study is subject to certain limitations. All experimental investigations were conducted on cement paste, and the direct quantitative applicability of the results to concrete containing aggregates, fibers, or reinforcement has not yet been experimentally validated. In heterogeneous cementitious systems, coarse aggregates, fibers, and reinforcement are expected to introduce additional scattering, anisotropy, and multi-path propagation effects, which can significantly influence ultrasonic wave attenuation and dispersion. Consequently, the current framework should be regarded as a matrix-level sensing and modeling approach that captures the intrinsic viscoelastic evolution of cementitious materials, rather than a complete description of full concrete or reinforced concrete systems.

Future work will focus on extending the proposed framework by incorporating effective medium theories, multi-scattering correction models, and guided-wave formulations to explicitly account for aggregate- and reinforcement-induced effects. In parallel, efforts will be directed toward integrating the sensing model into embedded ultrasonic sensor networks and automated construction platforms, as well as developing real-time parameter inversion algorithms for field deployment. These extensions will enable the transition from controlled material-scale sensing to component- and structural-scale SHM, further supporting lifecycle monitoring and digital twin applications for concrete infrastructure.

## Figures and Tables

**Table 1:** Chemical composition of P·O 42.5 cement (mass fraction).

Loss on Ignition (LOI)	SiO_2_	Al_2_O_3_	Fe_2_O_3_	CaO	MgO	SO_3_	Others	Total
1.62	26.38	9.61	4.34	50.09	3.16	2.01	2.79	100

Note: The data are presented as mass percent. The value for “Others” includes the total content of minor oxides (e.g., K_2_O, Na_2_O, TiO_2_, P_2_O_5_) and analytical uncertainty. The measured LOI (1.62%) complies with the standard requirement of ≤4% for Portland cement.

**Table 2:** Mean relative percentage error (MRPE) of different methods compared with experimental data.

W/C	Theoretical Model (%)	DFL Method (%)	Fractal Modification Model (%)	Kelvin-Voigt Model (%)
0.40	1.85	10.61	8.07	18.42
0.45	3.33	12.32	10.88	10.84
0.50	3.54	10.97	12.09	9.27
0.55	7.11	10.71	13.16	10.76

## Data Availability

Data are contained within the article.

## References

[1] Punurai W., Jarzynski J., Qu J., Kim J.-Y., Jacobs L.J., Kurtis K.E. (2007). Characterization of multi-scale porosity in cement paste by advanced ultrasonic techniques. Cem. Concr. Res..

[2] Gómez V.G., Garcia A.C., Castillo J.G., Roig I.B., Rosado M.V.B., Bernabeu J.J.P. Optimized ultrasonic attenuation measures for internal sulphate attack monitoring in portland cement mortars. Proceedings of the 2017 IEEE International Ultrasonics Symposium (IUS).

[3] Karaiskos G., Deraemaeker A., Aggelis D.G., Van Hemelrijck D. (2015). Monitoring of concrete structures using the ultrasonic pulse velocity method. Smart Mater. Struct..

[4] Khatib N., Ouacha E.H., Banouni H., Faiz B. (2025). Application of a novel ultrasound technique for real-time characterization of alkali accelerating admixture effects on cement mortar hydration. J. Build. Pathol. Rehabil..

[5] Khatib N., Hitar M.E.H., Lotfi E.M., Ouacha E.H., Banouni H., Faiz B., Décultot D., Ousbih M. (2021). Monitoring early age behavior of mortar by ultrasound technique for environmental issues: Effects of sand grading and salinity of mixing water. Constr. Build. Mater..

[6] Reinhardt H.W., Grosse C.U. (2004). Continuous monitoring of setting and hardening of mortar and concrete. Constr. Build. Mater..

[7] Lee H.K., Lee K.M., Kim Y.H., Yim H., Bae D. (2004). Ultrasonic in-situ monitoring of setting process of high-performance concrete. Cem. Concr. Res..

[8] Bekkour K., Kherfellah N. (2002). Linear viscoelastic behavior of bentonite-water suspensions. Appl. Rheol..

[9] Palermo A., Marzani A. (2016). Limits of the kelvin voigt model for the analysis of wave propagation in monoatomic mass-spring chains. J. Vib. Acoust..

[10] Jiao D., Shi C., Yuan Q. (2019). Time-dependent rheological behavior of cementitious paste under continuous shear mixing. Constr. Build. Mater..

[11] Wang X., Subramaniam K.V. (2011). Ultrasonic monitoring of capillary porosity and elastic properties in hydrating cement paste. Cem. Concr. Compos..

[12] Failla G., Zingales M. (2020). Advanced materials modelling via fractional calculus: Challenges and perspectives. Philos. Trans. R. Soc. A.

[13] Mainardi F. (2022). Fractional Calculus and Waves in Linear Viscoelasticity: An Introduction to Mathematical Models.

[14] Ribeiro J.G.T., de Castro J.T.P., Meggiolaro M.A. (2021). Modeling concrete and polymer creep using fractional calculus. J. Mater. Res. Technol..

[15] Chen J., Gong L., Meng R. (2024). Application of fractional calculus in predicting the temperature-dependent creep behavior of concrete. Fractal Fract..

[16] Bouras Y., Zorica D., Atanacković T.M., Vrcelj Z. (2018). A non-linear thermo-viscoelastic rheological model based on fractional derivatives for high temperature creep in concrete. Appl. Math. Model..

[17] Zhang C., Zhu Z., Zhu S., He Z., Zhu D., Liu J., Meng S. (2019). Nonlinear creep damage constitutive model of concrete based on fractional calculus theory. Materials.

[18] Guo S., Dai Q., Sun X., Sun Y., Liu Z. (2017). Ultrasonic techniques for air void size distribution and property evaluation in both early-age and hardened concrete samples. Appl. Sci..

[19] Xu D., Chen H., Hu Y., Sun D., Du P., Liu P. (2023). Ultrasonic monitoring and property prediction of cement hydration with novel omnidirectional piezoelectric ultrasonic transducer. J. Build. Eng..

[20] Xiao M., Zhu Y., Min W., Ye F., Li Y., Ding X., Ma T. (2025). A Method for Identifying Hydration Stages of Concrete Based on Embedded Piezo-Ultrasonic Active Sensing Technology. Materials.

[21] Li Z., Yu J., Liu Y., Zhang X., Zhang B., Elmaimouni L. (2025). Determination of fractional order for layered viscoelastic materials in cement grouted resin anchor bolt by using guided wave technology. Mech. Syst. Signal Process..

[22] Darmon M., Khalid N., Ramaniraka M., Dorval V., Henault J.-M., Chaix J.-F. (2025). Modeling of ultrasonic velocity and attenuation for concrete inspection: A review. Ultrasonics.

[23] Luo X., Zhou Y., Yi W. (2025). Time-dependent axial deformation of concrete: Development of a fractional calculus-based constitutive model. Eng. Struct..

[24] Hayek M., El Bitouri Y., Bouarab K., Yahia A. (2025). Structural Build-Up of Cement Pastes: A Comprehensive Overview and Key Research Directions. Constr. Mater..

[25] Bouabdallah A., Abdelatif B., Bouabdellah M., Souad M., Abdelwahhab K. (2025). Development and performance evaluation of self-leveling sand concrete: Enhanced fluidity, mechanical strength, durability, and non-destructive analysis. Constr. Build. Mater..

[26] Khatir A., Capozucca R., Khatir S., Magagnini E., Benaissa B., Cuong-Le T. (2024). An efficient improved gradient boosting for strain prediction in near-surface mounted fiber-reinforced polymer strengthened reinforced concrete beam. Front. Struct. Civ. Eng..

[27] Han G., Su Y.-F., He R., Huang C., Kong Z., Lin G., Feng Y., Lu N. (2025). Real-time concrete strength monitoring using piezoelectric sensors and deep learning. Nat. Commun..

[28] Bekzhanova Z., Memon S.A., Kim J.R. (2021). Self-sensing cementitious composites: Review and perspective. Nanomaterials.

[29] (2017). Methods for Chemical Analysis of Cement.

[30] Wang N., Xing G., Zhu T., Zhou H., Shi Y. (2022). Propagating seismic waves in VTI attenuating media using fractional viscoelastic wave equation. J. Geophys. Res. Solid Earth.

[31] He G., Mao Y., Ni W. (2007). A new fractal modification of ultrasonic attenuation model for measuring particle size in mineral slurries. Int. J. Miner. Process..

